# Gene coexpression network analysis reveals a novel metabolic mechanism of *Clostridium acetobutylicum* responding to phenolic inhibitors from lignocellulosic hydrolysates

**DOI:** 10.1186/s13068-020-01802-z

**Published:** 2020-09-26

**Authors:** Huanhuan Liu, Jing Zhang, Jian Yuan, Xiaolong Jiang, Lingyan Jiang, Zhenjing Li, Zhiqiu Yin, Yuhui Du, Guang Zhao, Bin Liu, Di Huang

**Affiliations:** 1 State Key Laboratory of Food Nutrition and Safety, Tianjin University of Science & Technology, Tianjin, 300457 China; 2 Key Laboratory of Food Nutrition and Safety, Tianjin University of Science & Technology, Ministry of Education, Tianjin, 300457 China; 3 TEDA Institute of Biological Sciences and Biotechnology, Tianjin Key Laboratory of Microbial Functional Genomics, Nankai University, Tianjin, 300457 China; 4 Institute of Bioenergy and Bioprocess Technology, Chinese Academy of Sciences, Qingdao, Qingdao, 266101 China

**Keywords:** Phenolic compounds, *Clostridium acetobutylicum*, Weighted gene co-expression network analysis, RNA sequencing, Acetone-Butanol-Ethanol, Lignocellulosic hydrolysates

## Abstract

**Background:**

Lignocellulosic biomass is a promising resource of renewable biochemicals and biofuels. However, the presence of inhibitors existing in lignocellulosic hydrolysates (LCH) is a great challenge to acetone-butanol-ethanol (ABE) fermentation by *Clostridium acetobutylicum*. In particular, phenolic compounds (PCs) from LCH severely block ABE production even at low concentrations. Thus, it is urgent to gain insight into the intracellular metabolic disturbances caused by phenolic inhibitors and elucidate the underlying mechanisms to identify key industrial bottlenecks that undermine efficient ABE production.

**Results:**

In this study, a time-course of ABE fermentation by *C. acetobutylicum* in the presence of four typical PCs (syringaldehyde, vanillin, ferulic acid, and *p*-coumaric acid) was characterized, respectively. Addition of PCs caused different irreversible effects on ABE production. Specifically, syringaldehyde showed the greatest inhibition to butanol production, followed by vanillin, ferulic acid, and *p*-coumaric acid. Subsequently, a weighted gene co-expression network analysis (WGCNA) based on RNA-sequencing data was applied to identify metabolic perturbations caused by four LCH-derived PCs, and extract the gene modules associated with extracellular fermentation traits. The hub genes in each module were subjected to protein–protein interaction analysis and enrichment analysis. The results showed that functional modules were PC-dependent and shared some unique features. Specifically, *p*-coumaric acid caused the most extensive transcriptomic disturbances, particularly affecting the gene expressions of ribosome proteins and the assembly of flagella, DNA replication, repair, and recombination; the addition of syringaldehyde caused significant metabolic disturbances on the gene expressions of ribosome proteins, starch and sucrose metabolism; vanillin mainly disturbed purine metabolism, sporulation and signal transduction; and ferulic acid caused a metabolic disturbance on glycosyl transferase-related gene expressions.

**Conclusion:**

This study uncovers novel insights into the inhibitory mechanisms of PCs for the first time and provides guidance for future metabolic engineering efforts, which establishes a powerful foundation for the development of phenol-tolerant strains of *C. acetobutylicum* for economically sustainable ABE production with high productivity from lignocellulosic biomass.

## Background

The exacerbation of global economic, social, and environmental problems due to the exhaustion of petroleum resources renders the production of renewable energy from low-cost plant biomass increasingly important, which has aroused intensive attention from governments and researchers worldwide [1, 2]. Lignocellulose, the most abundant renewable and low-cost biomass, is a promising substrate for biofuel production. Lignocellulose can be converted into either butanol or ethanol, and among these second generation vehicle biofuels, butanol displays better performance [3–5] due to its higher energy density, higher combustion heat, better engine compatibility, and decreased corrosivity [2].

Lignocellulosic feedstocks are usually pretreated with acids, bases, steam, and other harsh conditions, changing their physical properties to overcome the “recalcitrance” of lignocellulosic biomass, prior to utilization by microorganisms such as *Clostridium* spp. [6, 7]. Subsequently, pretreated lignocellulosic biomass is hydrolyzed into microorganism-accessible monosaccharides (hexose and pentose) through enzymatic hydrolysis [8]. During pretreatment and hydrolysis, a wide variety of toxic compounds are released from the lignocellulosic material [9, 10]. The presence of these compounds inhibits cell growth, substrate utilization, and product synthesis, significantly depressing the subsequent butanol production efficiency [11]. Generally, these compounds are classified into three categories according to their sources and properties, furans (e.g., furfural, 5-hydroxymethyl-furfural), weak acids (e.g., formic acid, acetic acid), and phenolic compounds (PCs; e.g., phenol, catechol, ferulic acid, syringaldehyde, vanillin, coumarin, and *p*-hydroxybenzoic acid) [10, 12]. Among them, PCs are more toxic than furans to microorganisms such as *Escherichia coli* and *Saccharomyces cerevisiae* [13], and smaller molecular weight PCs show stronger cytotoxicity [14].

Lignocellulosic hydrolysate (LCH) PCs are mainly generated during lignin depolymerization [15], and their types and final concentrations vary greatly from milligrams to grams per liter of LCH [16–18], based on the source of feedstocks, as well as the technical protocols of pretreatment, detoxification, and enzymatic hydrolysis [19–21]. The effects of PCs on acetone-butanol-ethanol (ABE) fermentation have been well studied [22, 23], but their mechanisms of inhibition remain unelucidated because of their low concentrations, various molecular structures, complexity, and difficulty to quantify. Therefore, a systematic investigation on the inhibitory mechanisms of PCs is urgently needed. The recent development of various omics technologies provides the opportunity for systematic profiling of the metabolic response mechanisms of microorganisms under various conditions that could be used to eliminate bottlenecks affecting target product synthesis. Great efforts have been made to dissect the metabolic mechanisms of *C. acetobutylicum* using transcriptomics [24, 25], proteomics [26, 27], and metabolomics [28, 29], providing a technical basis for dissecting PC inhibitory mechanisms in this microorganism.

In this study, we used an integrated strategy based on RNA sequencing (RNA-seq) and weighted gene coexpression network analysis (WGCNA) to systematically dissect gene expression changes in *C. acetobutylicum* under different phenolic stress conditions. Syringaldehyde, vanillin, ferulic acid, and *p*-coumaric acid were selected as representative LCH-derived PCs. Using WGCNA, genes with similar expression patterns were clustered and the association between modules and specific traits or phenotypes were analyzed [30, 31]. Our results promote fundamental understanding of the genetic regulatory mechanisms underlying *C. acetobutylicum*’s responses to PCs. We also propose novel potential metabolic engineering targets involved in regulating resistance to PC inhibitor stress. The transcriptome-guided approach demonstrated here could be a promising strategy to improve complex phenotypes in wild-type strains.

## Results

### Effect of different PCs on fermentation characteristics of *C. acetobutylicum*

**Fig. 1:**
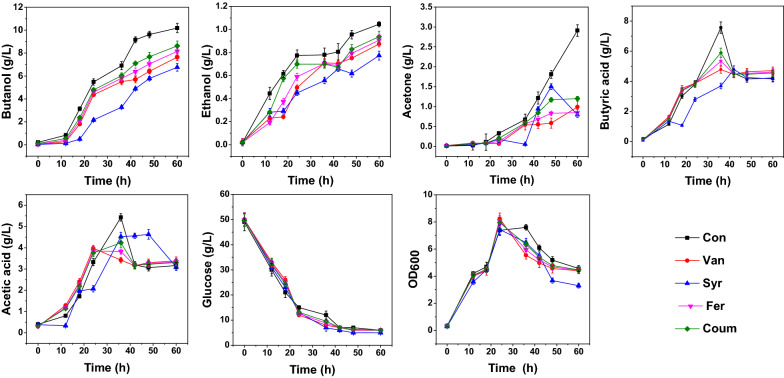
Time courses of ABE fermentation by *C. acetobutylicum* in the presence of four PCs. Each PC was added 12 h after the beginning of fermentation. Con, Van, Coum, Syr, and Fer denote the control, vanillin, *p*-coumaric acid, syringaldehyde, and ferulic acid samples, respectively. Error bars represent the standard deviation of three biological replicates

In this study, PCs (vanillin (Van), *p*-coumaric acid (Coum), syringaldehyde (Syr), and ferulic acid (Fer)) were added during the logarithmic growth phase (12 h, Fig. 1). The biomass (measured by assaying the optical density at 600 nm (OD_600_)) displayed no significant changes between samples from 12 to 24 h, when it peaked. The highest OD_600_ was 8.24 (Van), approximately 12% higher than in the control sample, while the lowest OD_600_ (7.45) occurred with Syr. However, the ethanol, acetone, and butanol production of the PC-treated samples were lower than those in the control sample at 60 h. Among the PC-treated samples, addition of Syr resulted in the greatest inhibition of butanol production (66.4% of the control), followed by Van (72.7% of the control), indicating that these PCs had notable impacts on fermentation performance.

The acetic acid and butyric acid concentrations peaked at 36 h, when these concentrations were much higher in the control sample than in the PC-treated samples (Fig. 1). The concentrations of these two organic acids subsequently decreased rapidly in the control sample, such that there were no significant differences between the samples at the end of fermentation, indicating that the acetic acid and butyric acid in the control sample were largely reabsorbed and utilized in the later stage of fermentation, promoting ABE synthesis [24, 32].

To compare short-term changes of fermentation characteristics caused by PC stress, a change ratio (CR) was used to express the ratio of production at 18 h compared to that at 12 h. As shown in Table 1, the CRs of acetone, and butyric acid in the treated samples were much lower than in the control sample, suggesting that the PCs negatively impacted acetone and butyric acid production. In addition, the CRs of acetic acid and the OD_600_ with Syr, and ethanol with Fer and Coum, were greater than in the control sample. Interestingly, butanol CRs were larger in the treated samples than in the control sample, which suggested that the addition of PCs actually promoted butanol production in the short term. Taken together, the results indicate that the PCs exert different effects on the fermentation products.

**Table 1:** Change ratios of fermentation products from 12–18 h

	Con	Van	Syr	Fer	Coum
Acetone	4.29	− 0.18	0.72	0.08	0.23
Ethanol	0.38	0.04	0.04	0.94	1.03
Butanol	2.74	5.64	3.55	4.38	3.35
Acetic acid	1.13	0.87	4.90	0.91	0.92
Butyric acid	1.62	1.14	− 0.20	1.21	1.25
Glucose	− 0.30	− 0.21	− 0.26	− 0.23	− 0.24
OD_600_	0.12	0.10	0.25	0.10	0.11

*Change ratio = (Value_18h-Value_12h)/Value_12h. OD_600_, optical density at 600 nm. Con, Van, Coum, and Fer denote the control, vanillin, *p*-coumaric acid, syringaldehyde and ferulic acid samples, respectively

### RNA-seq analysis of PC-treated *C. acetobutylicum*

To further analyze the relationships between PC stresses and intracellular metabolic disturbances, we performed a transcriptomic analysis, in which 18 h samples treated with each PC were harvested and subjected to RNA-sequencing.

To analyze the relationship between gene expression profiles and fermentation traits, identify highly synergistic gene sets and candidate biomarker genes or metabolic targets, the transcriptomic data were used for WGCNA model construction according to gene set connectivity [33, 34].

### WGCNA model construction

#### Soft threshold determination and network topology analysis of adjacency matrices based on WGCNA

**Fig. 2:**
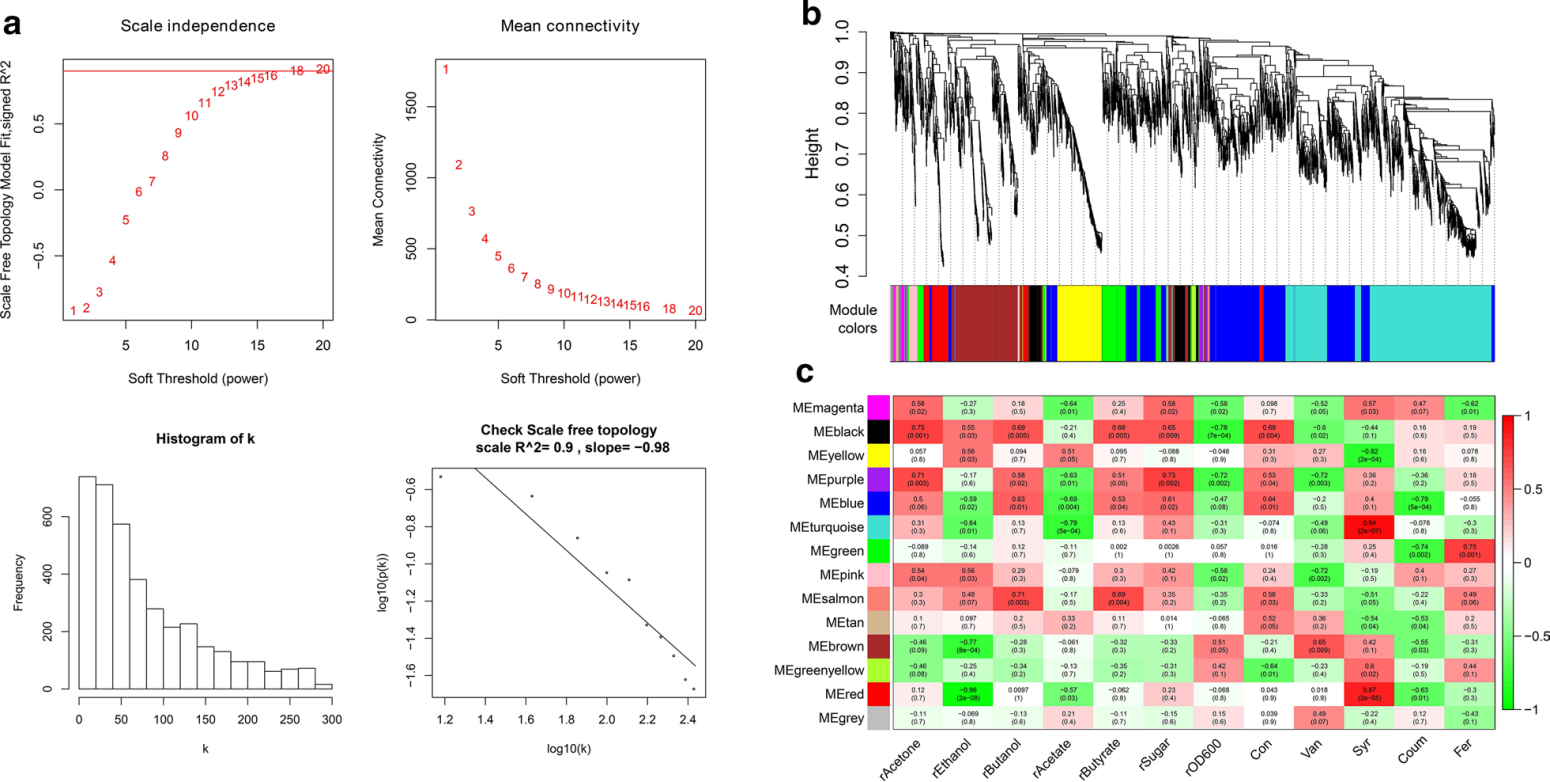
Construction of the WGCNA model. **a** Network topology analysis for adjacency matrices with different soft threshold powers. Red numbers indicate the soft‐threshold power corresponding to the correlation coefficient square value and mean connectivity. The linear model fit (R^2^) between log(p(k)) and log(k) was calculated from each adjacency matrix, where k = the connectivity and p(k) = the proportion of genes with connectivity k. **b** Clustering dendrogram of all expressed genes. Each row corresponds to a module eigengene and each column to a fermentation phenotype. **c** Module-traits relationships identified by WGCNA. Each cell contains the corresponding correlation in the first line and the *p*-value in the second line. Modules are colored as in the legend. Green and red denote negative and positive correlations, respectively. The grey module represents a collection of genes that could not be grouped into other modules

In WGCNA, the soft-threshold process transforms the correlation matrix to generate a series of adjacency matrices that mimics the scale-free topology, a phenomenon observed in gene expression networks and in a variety of complex biological systems in which the distribution of gene relationships follows a power decay law, i.e., genes with the highest numbers of connections occur least frequently [35]. A gradient method was applied to evaluate the scale-free fit index and the mean connectivity degree of different coexpression modules with power values ranging from 1 to 20. The optimal power value was 18 when the scale-free fit index was > 0.9 (Fig. 2a), meeting the requirements of WGCNA modeling and enabling further analysis. To ensure high reliability of the results, the minimum gene number of each module was set to 10.

#### Gene clustering and module-trait relationships

Figure 2b depicts the clustering dendrogram of all expressed genes. Based on clustering and dynamic pruning using the topological overlap measure (TOM), 3,911 correlated genes were clustered into 14 modules marked by different colors. Module-trait relationships were analyzed using correlations between the module eigengenes and 12 fermentation traits (specific increasing rates of Acetone, Butanol, Ethanol, Acetate, Butyrate, Sugar, OD_600_ as well as the conditions of Con, Van, Syr, Fer, and Coum), which enabled the identification of coexpression modules with significant correlations to fermentation traits. We considered the WGCNA modules with correction coefficients > 0.65 and *p *≤ 0.001 to be highly associated with fermentation traits, and identified 24 module-trait relationships (Fig. 2c).

**Fig. 3:**
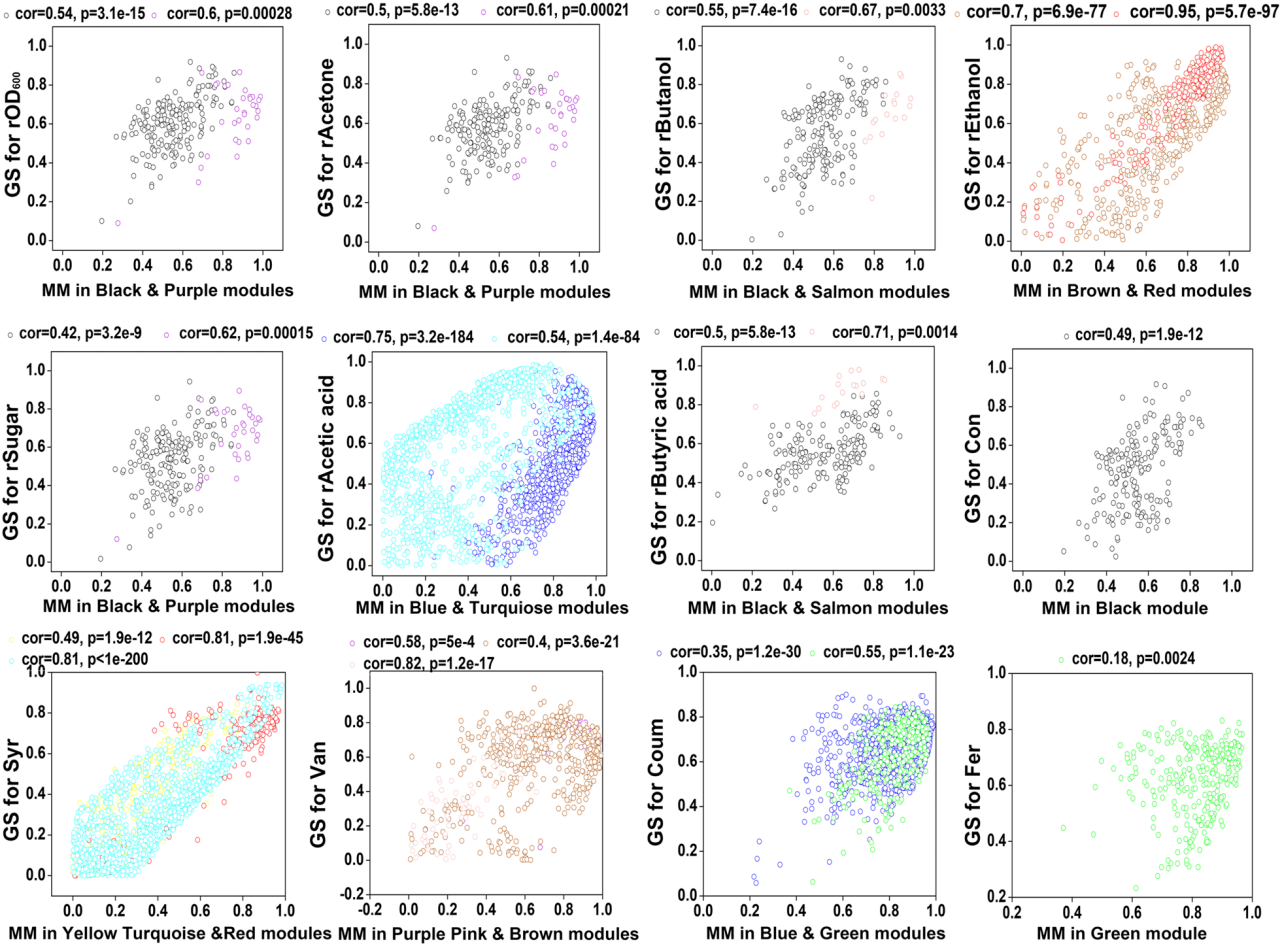
Module membership (MM) and gene significance (GS) in selected modules. Each color represents a selected WGCNA module. In each plot, the y-axis represents the GS of a fermentation trait and the x-axis represents the MMs of selected modules that were highly associated with those traits (correction coefficient  > 0.65 and *p* ≤ 0.001)

For each gene expression profile, the gene significance (GS) was calculated as the absolute value of the correlation between the expression profile and each external trait; and the module membership (MM) was defined as the correlation between the expression profile and each module eigengene. By calculating GS and MM values, genes that are highly significant for each trait and have high MMs in interesting modules can be identified. Scatterplots of GS vs. MM in each module are shown in Fig. 3 and additional details on the GS and MM are provided in Table SII (Additional file 1). GS and MM were correlated, illustrating that genes that were significantly associated with a fermentation trait were also important elements of modules.

### Functional enrichment analysis of hub genes

**Fig. 4:**
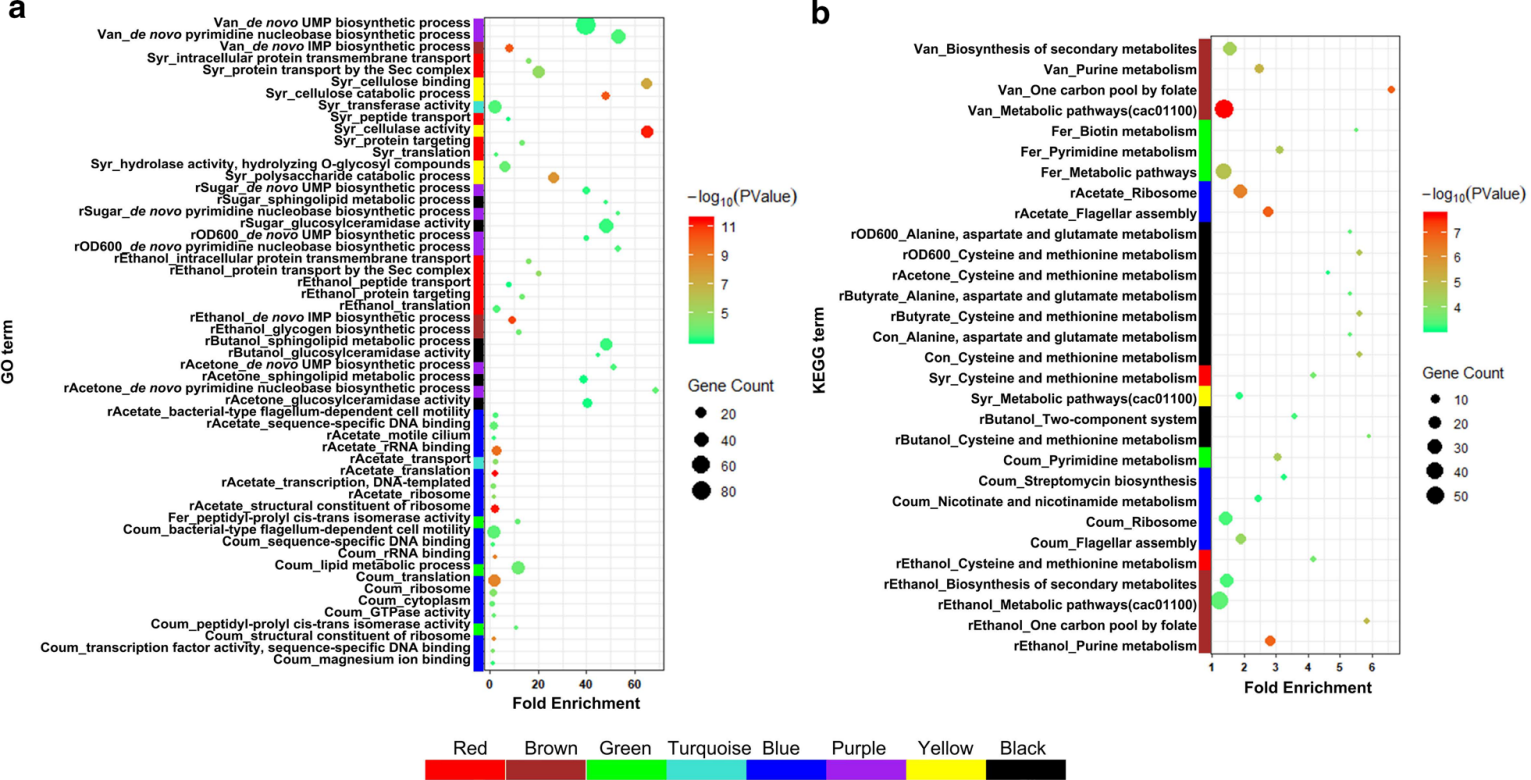
GO (**a**) and KEGG (**b**) enrichment based on the hub genes in each module. The enriched items with FDR < 0.05 were acceptable

Hub genes located in their respective modules, have a high likelihood of being critical components, and are representative of the module’s overall function within the network. In this study, genes with GS and MM values both > 0.5 were defined as the hub genes in their respective modules. WGCNA modules, related traits, eigengene counts, and hub genes are summarized in Table 2. Hub genes in each WGCNA module that were highly associated with the fermentation traits were subjected to gene ontology (GO) and Kyoto Encyclopedia of Genes and Genome (KEGG) pathway enrichment analysis with the Database for Annotation, Visualization and Integrated Discovery (DAVID). The results are shown in Fig. 4 and a further dissection is carried out as below.

**Table 2:** Summary of the relationships between traits and WGCNA modules

Trait	WGCNA module	Hub gene count/total gene count	Effect (Trait vs. module)
rButanol	Black	83/183	+
	Salmon	16/17	+
rButyrate	Black	108/183	+
	Salmon	14/17	+
rSugar	Black	80/183	+
	Purple	27/32	+
rAcetone	Black	94/183	+
	Purple	24/32	+
rOD_600_	Black	100/183	–
	Purple	27/32	–
rAcetate	Blue	590/1016	–
	Turquoise	298/1104	–
rEthanol	Brown	299/514	–
	Red	152/190	–
Con	Black	70/183	+
Coum	Blue	911/1016	–
	Green	227/282	–
Fer	Green	225/282	+
Syr	Yellow	49/296	–
	Turquoise	196/1104	+
	Red	152/190	+
Van	Purple	27/32	–
	Pink	10/68	–
	Brown	323/514	+

^*^ The table shows WGCNA modules that were highly associated with the fermentation characteristics (correction coefficient  > 0.65 and *p* ≤ 0.001) and the gene counts in each module. The specific rates of acetone, butanol, ethanol, acetate, butyrate, sugar, and biomass are represented by rAcetone, rButanol, rEthanol, rAcetate, rButyrate, rSugar, and rOD_600_, respectively. Con, Van, Coum, Syr, and Fer denote the control, vanillin, *p*-coumaric acid, syringaldehyde, and ferulic acid samples, respectively. + Positive correlation, − negative correlation. Eigengenes in the magenta, tan, and green-yellow modules were not significantly related to any traits

#### Biomass (OD_600_) and sugar (glucose)

As shown in Fig. 2c, the specific growth rate (rOD_600_) was highly negatively associated with the purple and black modules, while the opposite trend was observed for the specific consumption rate of sugar (rSugar), indicating that sugar consumption supported biomass synthesis. Enriched GO terms for rOD_600_ genes were de novo pyrimidine nucleobase biosynthetic process (GO:0006207) and de novo UMP biosynthetic process (GO:0044205), while glucosylceramidase activity (GO:0004348), de novo pyrimidine nucleobase biosynthetic process (GO:0006207), sphingolipid metabolic process (GO:0006665), and de novo UMP biosynthetic process (GO:0044205) were enriched by the rSugar-related gene modules. Glucosylceramidase activity and the sphingolipid metabolic process are related to membrane lipid metabolic processes. Two KEGG metabolic pathways were enriched by the OD_600_-related gene modules, namely cysteine and methionine metabolism (cac00270) and alanine, aspartate, and glutamate metabolism (cac00250).

#### ABE production

The specific production rate of acetone (rAcetone) was positively associated with the purple and black modules. Four GO terms were enriched, and were consistent with those enriched in the sugar-related modules. In addition, the KEGG pathway cysteine and methionine metabolism (cac00270) was also enriched.

The specific production rate of butanol (rButanol) was positively associated with the black and salmon modules. Two GO terms (GO:0004348, glucosylceramidase activity; GO:0006665, sphingolipid metabolic process) and two KEGG pathways (cac00270, cysteine and methionine metabolism; cac02020, two-component system) were enriched in the black module; however, hub genes of the salmon module were not enriched for any GO terms or KEGG pathways. Five genes in this module (KDP operon transcriptional regulatory protein KdpE (CA_C3677), potassium-transporting ATPase subunit C (CA_C3680), methyl-accepting chemotaxis protein (CA_C3476), d-alanyl-d-alanine carboxypeptidase (CA_C3297), and anaerobic C4-dicarboxylate transporter (CA_C3500)) are involved in the two-component system, a signaling pathway that regulates many bacterial characteristics, such as virulence, pathogenicity, symbiosis, motility, nutrient uptake, secondary metabolite production, metabolic regulation, and cell division. These systems regulate physiological processes in response to environmental or cellular parameters and enable adaptation to changing conditions [36], consistent with the view that butanol production results in the deterioration of fermentation.

The specific rate value of ethanol (rEthanol) was negatively associated with the brown module. In this module, seven enriched GO terms were identified (GO:0005978, glycogen biosynthetic process; GO:0006189, de novo IMP biosynthetic process; GO:0006412, translation; GO:0006605, protein targeting; GO:0015833, peptide transport; GO:0043952, protein transport by the Sec complex; GO:0065002, intracellular protein transmembrane transport), as well as five enriched KEGG pathways (cac00230, purine metabolism; cac00270, cysteine and methionine metabolism; cac00670, one carbon pool by folate; cac01100, metabolic pathways; cac01110, biosynthesis of secondary metabolites).

In ABE metabolic pathways, ethanol is closely related to C2 compounds, such as acetic acid, acetyl-P, and acetyl-CoA in primary metabolism. Butanol is derived from butyric acid, butyryl-P, and butyryl-CoA. Acetone is the decarboxylated product of acetoacetic acid, a branch pathway in the synthesis of butyric acid. The critical genes that regulate ABE synthesis are contained in the *sol* operon [37]. However, module-trait relationship analysis demonstrated no obvious links between ABE production and known ABE pathways, suggesting that ABE synthesis may be subjected to complex metabolic regulation.

#### Organic acid production

The specific production rate of acetate (rAcetate) was negatively associated with the blue module (GO:0006412, translation; GO:0003735, structural constituent of ribosome; GO:0019843, rRNA binding; ribosome; GO:0005840, transcription, DNA-templated; GO:0043565, sequence-specific DNA binding; GO:0071973, bacterial-type flagellum-dependent cell motility; GO:0031514, motile cilium; cac02040, flagellar assembly; cac03010, ribosome) and the turquoise module (GO:0006810, transport), demonstrating that rAcetate was highly related to protein biosynthesis, flagellar assembly, and transportation. The specific production rate of butyrate (rButyrate) was positively associated with the black module (cac00270, cysteine and methionine metabolism and alanine; cac00250, aspartate and glutamate metabolism) and the salmon module (no enriched terms), which were involved in amino acid metabolism.

#### PC stresses

As shown in Fig. 2c, treatment with each PC corresponded to different WGCNA modules, indicating that these PCs caused different metabolic disturbance to the strain. In detail, the control condition was highly associated with the black module, with hub genes enriched in the KEGG pathways cysteine and methionine metabolism (cac00270) and alanine, aspartate, and glutamate metabolism (cac00250).

Van treatment was associated with the purple (GO:0006207, de novo pyrimidine nucleobase biosynthetic process; GO:0044205, de novo UMP biosynthetic process), pink (no enriched terms), and brown (GO:0006189, de novo IMP biosynthetic process; cac01100, metabolic pathways; cac00670, one carbon pool by folate; cac00230, purine metabolism; cac01110, biosynthesis of secondary metabolites) modules, suggesting effects on nucleic acid metabolism.

Syr treatment was associated with the yellow (GO:0030245,cellulose catabolic process; GO:0000272, polysaccharide catabolic process; GO:0008810, cellulase activity; GO:0030248, cellulose binding; GO:0004553, hydrolase activity, hydrolyzing O-glycosyl compounds; cac01100, metabolic pathways), turquoise (GO:0016740, glycosyl transferase activity), and red (GO:0043952, protein transport by the Sec complex; GO:0065002, intracellular protein transmembrane transport; GO:0006605, protein targeting; GO:0006412, translation; GO:0015833, peptide transport; cac00270, cysteine and methionine metabolism) modules, suggesting effects on cellulose catabolism and protein transport.

Coum treatment was negatively related to the blue (GO:0006412, translation; GO:0071973, bacterial-type flagellum-dependent cell motility; GO:0005840, ribosome; GO:0005737, cytoplasm; GO:0019843, rRNA binding; GO:0003735, structural constituent of ribosome; GO:0003700, transcription factor activity, sequence-specific DNA binding; GO:0003924, GTPase activity; GO:0043565, sequence-specific DNA binding; GO:0000287, magnesium ion binding; cac02040, flagellar assembly; cac03010, ribosome; cac00760, nicotinate and nicotinamide metabolism; cac00521, streptomycin biosynthesis) and green (GO:0006629, lipid metabolic process; GO:0003755, peptidyl-prolyl cis–trans isomerase activity; cac00240, pyrimidine metabolism) modules, suggesting that its addition causes metabolic perturbations related to protein synthesis, flagellar assembly, and lipid metabolism.

Fer treatment was positively associated with the green module (GO:0003755, peptidyl-prolyl cis–trans isomerase activity; cac01100, metabolic pathways; cac00240, pyrimidine metabolism; cac00780, biotin metabolism).

To deeply explore the internal characteristics of the gene modules, protein–protein interaction (PPI) analysis was carried out.

### PPI analysis of hub genes under different PC stresses

**Fig. 5:**
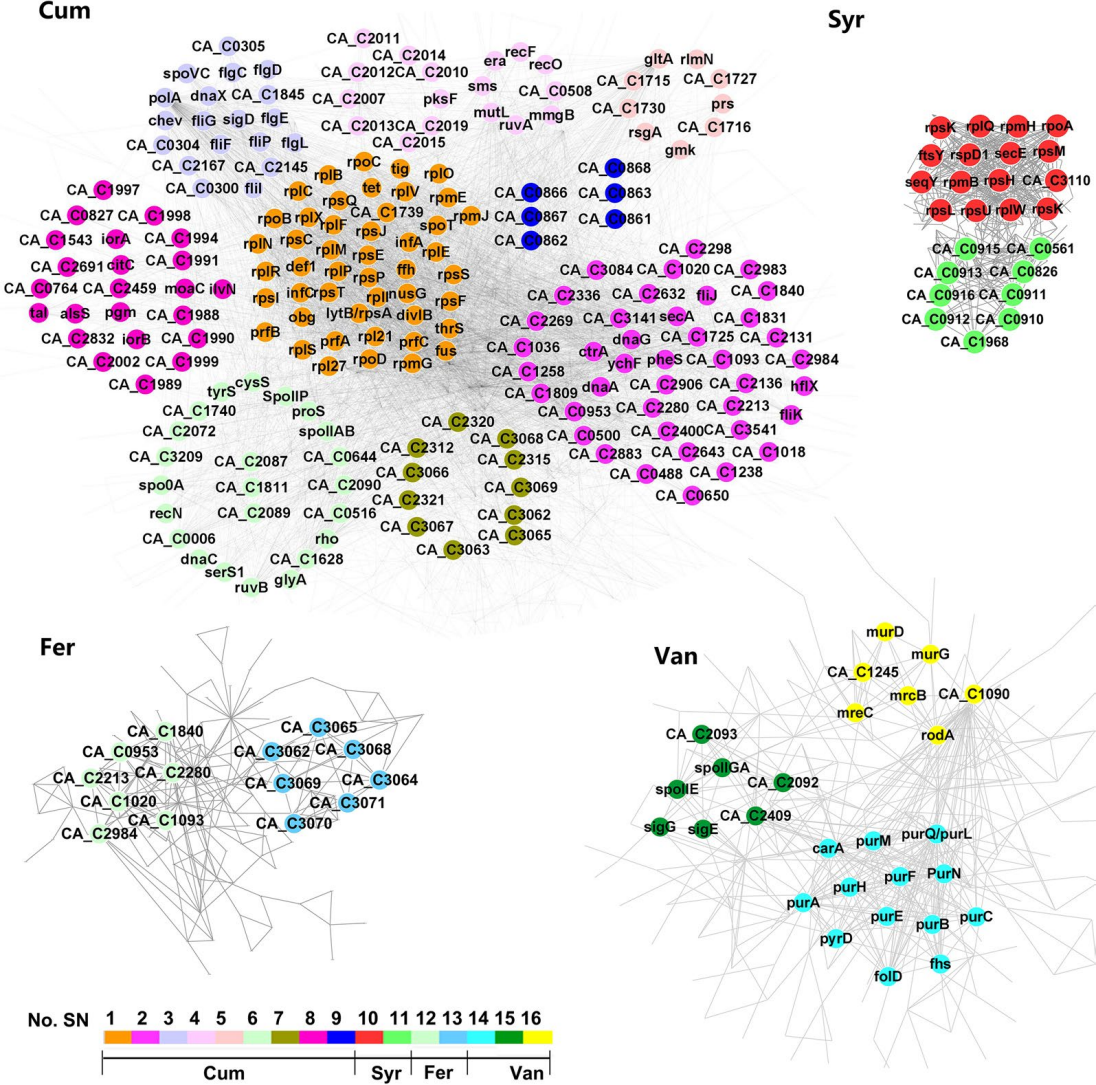
Protein-protein interactions between hub genes affected by each PC treatment. PPI networks of genes affected by **a** Coum, **b** Fer, **c** Syr, and **d** Van treatment. Each network contains hub genes from highly-associated WGCNA modules. Subnetworks extracted by MCODE are presented in different colors and numbered to differentiate them from other genes. Network nodes K-core value < 5 have been hidden

PPI networks represent webs of protein complexes formed by biochemical events and/or electrostatic forces that serve distinct biological functions while complexed. We performed the PPI analysis with the hub genes of all modules associated to each PC, obtaining the PC-specific interaction networks (Fig. 5). Using the STRING database, predicted functional associations between hub proteins with each treatment were identified based on known interactions (experimentally determined interactions from curated databases), predicted interactions (gene neighborhoods, gene fusions, and gene co-occurrences), and other evidence (text mining, coexpression, protein homology). As the result, sixteen high density subnetworks reveal the existence of highly interconnected gene sets that were biologically related to PC stress and were coexpressed, suggesting that they may play important roles under specific conditions. Information of the 16 subnetworks is listed in Table 3 and additional details regarding the PPI network are provided in Table SIII (Additional file 2).

**Table 3:** Enrichment of the PPI subnets (Coum, SN.1 ~ SN9; Syr, SN.10 ~ SN.11; Fer, SN.12 ~ SN.13; Van, SN.14 ~ SN.16)

Subnet	Enrichment item^a^	Function annotation	FDR
SN.1	BP GO:0044267	Cellular protein metabolic process	8.85E − 38
	MF GO:0003735	Structural constituent of ribosome	5.39E − 33
	CC GO:0005840	Ribosome	4.22E − 33
	KEGG cac03010	Ribosome	3.87E − 35
	UniProt KW-0687	Ribonucleoprotein	1.82E − 35
SN.2	BP GO:0006261	DNA-dependent DNA replication	4.78E − 02
SN.3	KEGG cac02040	Flagellar assembly	1.15E − 23
	UniProt KW-0282	Flagellum	5.41E − 17
SN.4	BP GO:0006281	DNA repair	6.64E − 06
	MF GO:0140097	Catalytic activity, acting on DNA	3.03E − 02
	CC GO:1990391	DNA repair complex	4.70E − 03
	KEGG cac03440	Homologous recombination	1.70E − 03
	UniProt KW-0234	DNA repair	2.15E − 07
SN.5	CC GO:0005737	Cytoplasm	2.03E − 02
	KEGG cac01230	Biosynthesis of amino acids	2.70E − 03
SN.6	BP GO:0090304	Nucleic acid metabolic process	8.50E − 04
	MF GO:0008144	Drug binding	8.97E − 05
	CC GO:0005737	Cytoplasm	9.30E − 03
	KEGG cac00970	Aminoacyl-tRNA biosynthesis	1.20E − 03
	UniProt KW-0030	Aminoacyl-tRNA synthetase	1.20E − 03
SN.7	KEGG cac00523	Polyketide sugar unit biosynthesis	6.80E − 04
	UniProt KW-0808	Transferase	5.10E − 03
SN.8	KEGG cac01130	Biosynthesis of antibiotics	3.56E − 06
	UniProt KW-0501	Molybdenum cofactor biosynthesis	2.06E − 02
SN.9	UniProt KW-0418	Kinase	6.50E − 04
SN.10	BP GO:0034645	Cellular macromolecule biosynthetic process	9.66E − 13
	MF GO:0003735	Structural constituent of ribosome	2.38E − 13
	CC GO:0005840	Ribosome	2.08E − 13
	KEGG cac03010	Ribosome	1.15E − 13
SN.11	KEGG cac00500	Starch and sucrose metabolism	5.14E − 06
	UniProt KW-0732	Signal	8.54E − 09
SN.12	None		
SN.13	UniProt KW-0808	Transferase	4.20E − 02
SN.14	BP GO:0009260	Ribonucleotide biosynthetic process	3.64E − 06
	MF GO:0016879	Ligase activity, forming carbon–nitrogen bonds	8.81E − 07
	KEGG cac00230	Purine metabolism	8.75E − 13
	UniProt KW-0658	Purine biosynthesis	1.76E − 17
SN.15	BP GO:0030435	Sporulation resulting in formation of a cellular spore	3.17E − 05
	MF GO:0016987	Sigma factor activity	5.00E − 04
	UniProt KW-0749	Sporulation	6.55E − 06
SN.16	BP GO:1901564	Organonitrogen compound metabolic process	2.30E − 03
	MF GO:0016758	Transferase activity, transferring hexosyl groups	6.30E − 03
	KEGG cac00400	Phenylalanine, tyrosine and tryptophan biosynthesis	5.52E − 05
	UniProt KW-0133	Cell shape	2.51E − 06

^a^The genes in each subnet were enriched in GO, KEGG and UniProt databases, and the items with minimum FDR (< 0.05) was listed

#### p-Coumaric acid-associated PPI subnetworks

A total of 990 hub genes from the blue and green modules of the WGCNA were used to construct the PPI network. Using the MCODE algorithm, nine dense regions with high internal connectivity (subnetworks SN-1 ~ SN-9 in Fig. 5) were identified. The genes in each subnetwork were enriched in GO, KEGG and UniProt databases, and the items with minimum FDR (< 0.05) was listed in Table 3. Enrichment analysis demonstrated that these subnetworks were associated with ribosome, DNA replication, flagellum, cytoplasm and biosynthesis of amino acids, kinase, glycosyl transferase, purine metabolism, sporulation and as well as several other cellular functions, suggesting wide effects on cellular metabolism caused by the addition of *p*-coumaric acid.

Interestingly, SN-4 contained 18 genes that could be divided into two subnetworks (Fig. 5), one associated with DNA damage and repair, the other including the neighborhood genes CA_C2007 ~ CA_C20015 and CA_C20019. Among them, glycosyl transferase (CA_C2007), 3-oxoacyl-ACP synthase (*pksF*, CA_C2008), 3-hydroxyacyl-CoA dehydrogenase (*mmgB*, CA_C2009), Fe-S oxidoreductase (CA_C2010), 3-oxoacyl-ACP synthase (*fabH*, CA_C2011), enoyl-CoA hydratase (*fadB*, CA_C2012), esterase (CA_C2014), and malonyl CoA-ACP transacylase (CA_C2019) are associated with the fatty acid biosynthetic process. In additions, we noted *spoT* and *obg*, which encode the (p)guanosine 3′-diphosphate 5′-diphosphate (ppGpp) synthetase and GTPase (binding GTP, GDP, and possibly (p)ppGpp with moderate affinity); in eubacteria ppGpp is a mediator of the response to changes in nutritional abundance, which coordinates a variety of cellular activities [38].

#### Syringaldehyde-associated PPI subnetworks

A total of 263 hub genes from the blue and green modules of WGCNA were used to construct the PPI network, and two dense regions (SN-10 and SN-11) were extracted. SN-10 contained 16 hub genes which were enriched in ribosomal proteins. SN-11 contained 9 hub genes, which were enriched in starch and sucrose metabolism and signal transduction. Further analysis showed that CA_C0910, CA_C0911, CA_C0912, CA_C0913, CA_C0915 and CA_C0916 encode the cellulosomal proteins. The cellulosome is a cellulase system that exist in cellulolytic microorganisms such as *C. thermocellum*, *C. cellulolyticum*, and *C. cellulovorans* [39]. Cellulase and hemicellulase form a multienzyme complex structure through an anchorage-adhesion mechanism. Cellulosomes are attached to the bacterial cell wall by cell adhesion proteins, but lack cellulolytic activity in *C. acetobutylicum* [40].

#### Ferulic acid-associated PPI subnetworks

A total of 156 hub genes from the blue and green modules of the WGCNA were used to construct the PPI network, and two dense regions (SN-12 and SN-13) with extensive internal connections were extracted (Table 2). SN-12 contained seven hub genes that were clustered because of gene co-occurrence, but lacked annotations or descriptions. SN-13 contained seven genes that clustered by gene neighborhood, among which CA_C3068, CA_C3069, CA_C3070, CA_C3071 were related to glycosyl transferase.

#### Vanillin-associated PPI subnetworks

A total of 241 hub genes from the blue and green modules of the WGCNA were used to construct the PPI network. Three dense regions (SN-14, SN-15, and SN-16) were extracted (Fig. 5). SN-14 contained 13 hub genes that were enriched for roles in purine metabolism. SN-15 contained seven hub genes that were enriched for roles in sporulation, which was considered to be associated with the deterioration of fermentation [41, 42]. SN-16 contained 17 hub genes which were involved with organonitrogen compound metabolic process, glycosyl transferase activity, phenylalanine, tyrosine and tryptophan biosynthesis and cell shape (Table 3).

## Discussion

PCs widely exist in LCH and have strong toxicity to microorganisms. For ABE production, several research groups have examined PC-induced inhibition of *Clostridia* [10, 43, 44]. Cho et al. [10] assessed the inhibitory effects of six PCs (*p*-coumaric acid, ferulic acid, 4-hydroxybenzoic acid, vanillic acid, vanillin, and syringaldehyde) on *C. beijerinckii*. At 1 g/L, the tested PCs inhibited cell growth by 64 ~ 74% and completely inhibited butanol production. Ezeji et al. [43] evaluated the impact of PCs on *C. beijerinckii* growth and butanol production, and found that > 0.3 g/L ferulic acid was most toxic to *C. beijerinckii* growth and completely inhibited butanol production, followed by syringaldehyde. Chen et al. [44] established a mathematical model to evaluate the inhibitory effects of phenolic derivatives on ABE fermentation by *C. saccharoperbutylacetonicum*.

In this study, due to the low doses used, the four PCs did not show significant inhibitory effects on biomass and sugar consumption, but caused different irreversible effects on ABE fermentation (Fig. 1), indicating that under these conditions, *C. acetobutylicum* survived by regulating intracellular metabolism to cope with environmental stress, but at the expense of fermentation performance.

Generally, ABE fermentation can be divided into two stages, acidogenesis and solventogenesis. In the acidogenesis stage, large amounts of acetic acid and butyric acid are generated, accompanied with biomass accumulation, while in the solventogenesis stage the organic acids are reabsorbed and utilized by the cells to produce ethanol and butanol. With respect to metabolic pathways, butyric acid and acetic acid are the precursors of butanol and ethanol, respectively, so the levels of these organic acids and their corresponding alcohols are closely related. In the WGCNA models (Fig. 2c), we found that butanol production was highly related to the black and salmon modules, which were linked with butyric acid production in the module-trait correlation analysis. However, hub gene functional enrichment analysis revealed differences. Butanol production was associated with the two-component system, sphingolipid metabolic process, and glucosylceramidase activity, which are involved in cellular stress responses, while butyric acid was related to alanine, aspartate, and glutamate metabolism.

Similarly, acetic acid production was closely related to flagellar assembly (cell mobility) and ribosome function (protein synthesis), while ethanol was related to specific secondary metabolic activities, such as the biosynthesis of glycogen and secondary metabolites. Moreover, biological functions closely related to acetone and butanol were similar. The sphingolipid metabolic process and glucosylceramidase activity are both related to cellular membrane lipid metabolism. These analyses indicate that as a systematic method for describing gene association patterns among different samples, WGCNA can be used to identify highly synergistic gene sets and identify candidate biomarker genes or metabolic targets by associating gene sets and phenotypes.

Some other microorganisms, e.g., *S. cerevisiae*, can convert PCs into less toxic compounds, achieving detoxification at the cost of reduced product synthesis [14], which was a in situ detoxification by the oxidoreductive pathway [45]. Larsson et al. [14] evaluated the influence of hydroxy-methoxy-benzaldehydes, diphenols/quinones, and phenylpropane derivatives on *S. cerevisiae* cell growth and ethanol formation. Aromatic alcohols were detected as the reduction products of their corresponding aldehydes. In another report, Shen et al. [46] reported upregulated oxidoreductase and antioxidant activities when *S. cerevisiae* was exposed to vanillin. However, in our study, no significant correlation between PCs and any oxidoreductive metabolism-related KEGG pathways, GO terms, or protein complexes were observed, indicating that the effects of PCs on *C. acetobutylicum* were distinct from their effects on *S. cerevisiae.*

Systems biology methods provides a global perspective on the effects of inhibitors on microbial metabolism. In this study, a WGCNA strategy based on RNA-seq transcriptomics was applied to identify the metabolic perturbations in gene expression caused by four LCH-derived PCs, and extract the metabolic modules associated with extracellular fermentation characteristics. Several gene sets that were highly associated with fermentation traits were identified and analyzed for enriched biological functions. Based on WGCNA, hub gene enrichment, and PPI analysis, four PCs exerted effects on cell metabolism via both different and shared targets.

**Fig. 6:**
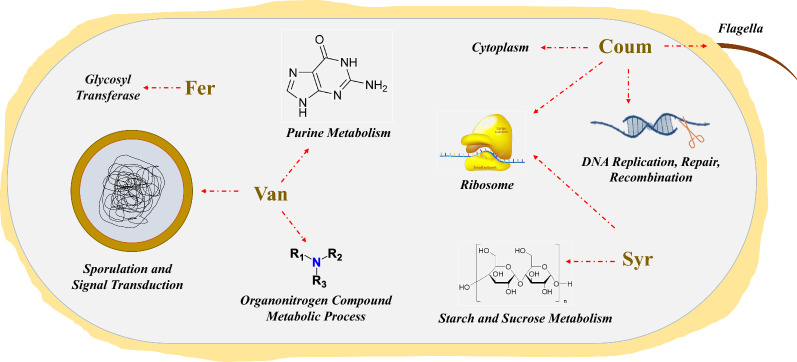
Metabolic response mechanism of *C. acetobutylicum* to lignocellulose-derived PCs

PCs had significant effects on important physiological processes. As shown in Fig. 6, a potential metabolic mechanism in response to lignocellulose-derived PCs stress was proposed. In general, *p*-coumaric acid has an effect mainly on the assembly of ribosome, flagella, DNA replication, repair, and recombination; syringaldehyde on ribosome protein gene expressions, starch and sucrose metabolism; vanillin mainly on purine metabolism, sporulation and signal transduction, organonitrogen compound metabolic process; ferulic acid mainly on the gene expression of glycosyl transferase. These results suggest that PCs may interfere with the fermentation profiles by different intracellular metabolic disturbances.

## Conclusions

In summary, we have performed an in-depth analysis of key pathway responses to various lignocellulose-derived PCs. Our results indicate that precise regulation of these pathways or bottleneck steps are essential for strain development and aid in the improved ABE production from LCH.

## Methods

### Media and strain cultivation

Spores of *C. acetobutylicum* ATCC 824 were bought from the American Type Culture Collection (ATCC, Maryland, USA) and stored at −80 °C. Reinforced *Clostridium* medium was used for seed cultivation [47]. Fermentation was performed in P2 medium containing 50 g/L glucose, 0.5 g/L K_2_HPO_4_, 2.2 g/L ammonium acetate, 0.5 g/L KH_2_PO_4_, 0.2 g/L MgSO_4_·7H_2_O, 0.01 g/L FeSO_4_·7H_2_O, 0.01 g/L NaCl, 0.01 g/L MnSO_4_·H_2_O, 1 mg/L *p*-aminobenzoic acid, 1 mg/L vitamin B1, and 0.001 mg/L biotin.

*C. acetobutylicum* spores were heat-shocked at 85 °C for 10 min and then inoculated at a 1% (v/v) volume size for seed cultivation in a YQX-II anaerobic incubator (Shanghai Longyue Instrument Equipment Co., Ltd., China) for 24 h (to an OD_600_ of ~ 3.0). Anaerobic ABE fermentation was performed in a 3.5 L fermenter (NBS BIOFLO-2000, New Brunswick Scientific Co., Inc., U.S.A) with a 10% (v/v) inoculation volume and a 2.0 L working volume. The stirring speed was maintained at 100 rpm and the temperature at 37 °C. Broth pH was monitored by pH electrode and maintained at 5.8 with 6 M ammonia and 6 M HCl.

### Addition of phenolic inhibitors

The four PCs used in this study (vanillin, *p*-coumaric acid, syringaldehyde, and ferulic acid), were added separately 12 h after the beginning of fermentation to 200 mg/L. The main fermentation parameters, OD_600_, glucose, acetic acid, butyric acid, ethanol, butanol, and acetone, were monitored as previously reported [47].

### Biomass and glucose measurements

The biomass was determined by ultraviolet spectrophotometry at 600 nm. Glucose was measured with a Bioanalyzer SBA 40D equipped with a biosensor (Biology Institute of Shandong Academy of Sciences, China) according to the manufacturer’s instructions.

### Fermentation product measurements

Ethanol, acetone, butanol, acetic acid, and butyric acid were analyzed with a gas chromatograph [48] (GC, 430-GC, Bruker Daltonics Inc., USA) equipped with a BW-SWAX capillary column (30 m × 0.32 mm ID × 0.25 m) and an FID detector. The heating procedure was 80 °C for 2 min, then increases of 10 °C/min for 2 min and 50 °C/min for 2 min, then 230 °C for 2 min. The inlet and detector temperatures were both 250 °C. The split ratio was 1:20, and the ratio of carrier gas (nitrogen): supplementary gas (nitrogen): air: hydrogen = 1:20:30:35 mL/min.

### RNA-seq and quantification of gene expression levels

A 10 mL sample of each fermentation broth after 18 h was used for RNA-seq. Total RNA was extracted using TRIzol Reagent (Invitrogen, Carlsbad, USA) according to the manufacturer’s instructions, and purified using an RNeasy Mini Kit (Qiagen, Germany) including an on-column DNase (Qiagen) digestion step to avoid contamination with genomic DNA. Sequencing libraries were generated using NEBNext^®^ Ultra™ Directional RNA Library Prep Kit for Illumina^®^ (New England Biolabs Ltd., USA) following the manufacturer’s recommendations, and index codes were added to attribute sequences to each sample. Clustering of the index-coded samples was performed on a cBot Cluster Generation System using a TruSeq PE Cluster Kit v3-cBot-HS (Illumina, Inc., USA). After cluster generation, library preparations were sequenced on an Illumina Hiseq platform and paired-end reads were generated. The total number of raw reads obtained from the samples (Con, Van, Syr, Fer, and Coum) ranged from 9.18 to 11.37 million. Raw data in fastq format were first processed to obtain clean data by removing reads containing adapters and poly-N tracts and low-quality reads from the raw data. At the same time, the Q20, Q30, and GC content of the clean data were calculated. Clean reads were mapped to the *C. acetobutylicum* ATCC 824 genome (NC_003030.1) and plasmid pSOL1 (NC_001988.2; NCBI, https://www.ncbi.nlm.nih.gov/genome/?term=ATCC+824) using Bowtie 2 v2.2.3 [49]. HTSeq v0.6.1 was used to count the read numbers mapped to each gene. Then, the expected number of FPKMs (fragment per kilobase of transcript sequence per millions base pairs sequenced) of each gene was calculated to determine their expression values, based on the length of each gene and the read counts mapped to it [50]. 3,911 genes in each sample had  ≥ 1 (FPKM) and were considered expressed (Table SI in Additional file 3).

### Construction of the WCGNA network

The WGCNA model was built from the transcriptome datasets and the FPKM by calculating weighted Pearson correlation matrices relative to the FPKM, according to the R 3.5.1 online tutorial (https://horvath.genetics.ucla.edu/html/CoexpressionNetwork/Rpackages/WGCNA/Tutorials/). Briefly, a matrix was constructed by calculating Pearson correlations to measure the similarity between gene expression profiles of different samples. Then, the similarity matrix was transformed into an adjacency matrix raised to a β exponent (soft threshold) based on the free-scale topology criterion. The TOM was used to define modules based on dissimilarity (1-TOM). Modules were merged based on dissimilarity between their eigengenes, which are first principal components of each module and represent the gene expression profiles within them [30]. Genes with highly similar correlation relationships were grouped into the same modules through hierarchical clustering based on the TOM results. Each gene module was assigned a color, with genes not sorted to any specific module grouped in grey. Module-trait associations were estimated using the correlation between the module eigengene and rAcetone, rButanol, rEthanol, rAcetate, rButyrate, rSugar, and rOD_600_ values based on the fermentation characteristic curves, as well as the control, syringaldehyde, vanillin, ferulic acid, and *p*-coumaric acid treatment conditions, allowing the identification of modules highly correlated with both fermentation traits and treatments. Genes in modules with significant module-trait associations (coefficient  > 0.65 and *p* value < 0.001) were included in functional enrichment analysis.

### Hub gene determination

Using the GS and MM, genes with high significance for each trait and high MMs in interesting modules can be identified. The intramodular connectivity was computed for each gene by summing the strengths of its connections with other module genes and dividing this number by the maximum intramodular connectivity. Genes with maximum intramodular connectivity were regarded as intramodular hub genes [51], which had GSi > 0.5 and MMi > 0.5.

### GO and KEGG enrichment analysis

GO and KEGG enrichment analysis of hub genes was performed in DAVID [52] (https://david.ncifcrf.gov). Enriched terms with *p*-values<0.05 were considered significant and used for biological function annotation.

### PPI analysis

PPI analysis was performed in STRING [53] (https://string-db.org) with default parameters. Cytoscape v3.6.1 [54] was used to depict the gene interaction network. Molecular Complex Detection (MCODE) [55] is a Cytoscape plug-in that detects densely connected regions in large PPI networks that may represent molecular complexes, and was used to extract the core subnetworks, with a K-core value  > 5.

Unless otherwise specified, all reagents used in this study were purchased from Sigma-Aldrich Co. Ltd. and were of  > 98% purity. Each experiment was repeated at least three times.

## Supplementary information

**Additional file 1:** Table SII. GS & MM in WGCNA Modules.

**Additional file 2:** Table SIII. PPI and Subnetwork.

**Additional file 3:** Table SI. FPKM and Gene Annotation.

## Data Availability

The datasets used and/or analyzed during the current study are available from the corresponding author on reasonable request.

## Abbreviations

ABE: Acetone-Butanol-Ethanol
PC: Phenolic compound
LCH: Lignocellulosic hydrolysates
WGCNA: Weighted gene co-expression network analysis
PPI: Protein-protein interaction
FPKM: Fragments per kilobase of transcript sequence per million base pairs sequenced
TOM: Topological overlap matrix
GS: Gene significance
MM: Module membership
CR: Change ratio
SN: Subnetwork
RNA-Seq: RNA sequencing
